# Editorialets

**Published:** 1909-07

**Authors:** 


### Editorialets.

Dr. W. H. Welch of Baltimore was elected President. Next meeting, St. Louis.

The hook worm is reported to have made its appearance in Southern Texas. The State Board of Health is "going for" him with a sharp stick.

Dr. E. B. Parsons of Palestine has been appointed a member of the State Board of Health, vice Dr. H. E. Whitesides, resigned, on account of ill health. An excellent selection.

The Atlantic City Meeting, A. M. A.—The Indiana Tentacle collar editor says: "Amid the constant lashing of the waves the American Medical Association met and adjourned." Funny.

---

Dr. J. F. Bailey, the Osteopath member of the State Board of Medical Examiners, has removed from Waco to Austin. Dr. Jno. T. Moore has removed from Galveston to Houston and opened up a sanitarium in copartnership with Dr. Pritchell and Dr. (Miss) M. A. Wood.

---

The law in Texas against giant crackers and toy pistols went into effect June 12 (ult.). It imposes a tax of $500 a year on the sale of these deadly things. Dealers were caught with large supplies on hand. It is now worthless, but there is no estimating how many boys' lives will be saved by it.

---

Dr. C. E. Cantrell of Greenville was elected a trustee of the American Medical Association, vice Happell, deceased, at the recent meeting at Atlantic City. Dr. Cantrell is also president of the Judicial Council, A. M. A. Dr. Frank Paschal of San Antonio was also appointed chairman of the Committee on Amendments to the Constitution, A. M. A.

---

A movement is on to establish a $250,000 sanatorium for consumptives at San Angelo, to be a joint stock company. Shares, $100. Two hundred and fifty acres of ground are to be purchased. Bungalows for patients instead of tents. Fifty thousand dollars of the stock was promptly taken by the San Angeloans. It should be a paying investment. Mr. Hulbut is agent and is calling on Texas physicians.

---

What's the matter? When the A. M. A. met in Chicago in 1908 there were 6646 present; at Boston in 1906, 4722; Atlantic City in 1907, 3713. At Atlantic City, 1909, 3090. That is less than half the attendance at Chicago; less by 33.3 per cent than at Boston, and less by 623 than at Atlantic City two years ago. Why this falling off in attendance, when the record shows an increase in membership year by year? The attendance this year represents about 8 per cent of its membership.

---

We learn that at the Atlantic City meeting of the A. M. A. organization of the American Medico-Chirurgical Association was effected, with many prominent physicians as charter members. No further particulars to date. We also learn that the A. M. A. sustained Dr. Wiley and repudiated the findings of the Remsen Board with reference to the use of benzoate of soda as a food preservative, and that Dr. Reed of Cincinnati was appointed to convey the information to Mr. Taft. (Good thing, perhaps, that he does not have to tell Mr. Roosevelt.)

---

DR. GEO H. LEE of Galveston has been elected by the Board of Regents of the University of Texas to the chair of gynecology and obstetrics in the University Medical College, vice Prof. J. F. Y. Paine, resigned, effective in September, 1910, when he becomes emeritus professor of those branches. This is a most excellent selection and the THE JOURNAL congratulates the Board on its discriminating good sense and judgment; the Faculty and the students on the acquisition of so able and popular a confrere, and shakes hands with itself and rejoices in its friend's advancement.

---

VIVE LE ROI! Dr. Geo. H. Simmons, the Poo-Bah and First Lord of the A. M. A., "our peerless leader," as certain toadies style him, advertising specialist in that "filthiest of diseases" denounced in others by the Kentucky Tentacle Mac, guaranteeing "a cure in every case, no matter of how long standing," was reelected at Atlantic City, given an ovation and presented with a gold watch. Dr. Cantrell got up the subscription to buy the watch. If after Galveston there was a shadow of a doubt who is running our machine, and where we are "at," all doubt is now removed. Ephraim is wedded to his idols; let him alone.

Bow, wow, wow, whose dog art thou?
I'm Tom Tucker's dog; whose dog art thou?

---

### Letter to the Editor.

GAINESVILLE, TEXAS, June 5, 1909.

*Dr. F. E. Daniel, Austin, Texas.*

MY DEAR DOCTOR: It was impossible for me to get to Galveston, but from what I have heard and read a pretty hot time was enjoyed regardless of the Gulf breeze. This is to assure you I am with you in your fight for a white man's association, composed only of regular physicians, and it occurs to me it will be wise to have this matter thoroughly worked out in the county societies and instructions given the delegate to the Dallas meeting saying vote for or vote against the Daniel amendment. The fight can and should be won long before the Dallas meeting. I feel assured the majority of the local societies over the State will instruct their delegate to vote for your amendment.

Very truly,

C. R. Johnson.

